# Entropy-Constrained M^2^ANet for Early Fault Prediction of Wind Turbines

**DOI:** 10.3390/e28060666

**Published:** 2026-06-11

**Authors:** Jingchan Lv, Zhihai Yao

**Affiliations:** 1 Department of Physics, Changchun University of Science and Technology, Changchun 130022, China; ljingchan@cust.edu.cn; 2 College of Physics and Electronic Information, Baicheng Normal University, Baicheng 137000, China

**Keywords:** wind turbine, fault prediction, SCADA data, M^2^ANet, imbalanced learning, multi-system faults

## Abstract

Early fault prediction of wind turbines is critical for ensuring wind farm safety and reducing operation and maintenance costs. However, the latent and progressive nature of incipient faults, together with concurrent failures across multiple subsystems, makes accurate root-cause identification challenging. In addition, severe class imbalance between normal and faulty samples further degrades prediction performance, particularly for minority fault types. To address these challenges, this paper proposes a novel fault prediction model, M^2^ANet, using SCADA data within a 30-min pre-fault window. The model combines a dual-memory module with progressive dilated convolutions to efficiently capture multi-scale temporal dependencies from high-dimensional operational variables. An entropy-bias penalty is further introduced into the loss function to adaptively regularize the predicted probability distribution, alleviating overconfidence under imbalanced data conditions and improving the recognition of minority faults. Experiments on a real-world wind farm dataset show that M^2^ANet achieves an overall accuracy of 90.73% and a weighted F1-score of 90.62% in multi-class fault prediction, outperforming 10 representative baseline models. In addition to these aggregate metrics, per-class evaluation confirms the model’s robustness under class imbalance. Notably, for yaw system faults, which account for only 1.9% of the samples, M^2^ANet achieves a recall of 95.92% with a 30-min-ahead warning. These results demonstrate its effectiveness and reliability for early fault prediction in practical wind turbine applications.

## 1. Introduction

Wind energy, as a mature and environmentally friendly renewable energy source, has experienced rapid growth in both installed capacity and deployment scale in recent years [1]. However, wind turbines are structurally complex and are often installed in remote onshore areas or offshore environments with harsh operating conditions, making continuous condition monitoring challenging. As a result, operation and maintenance costs account for approximately 2–30% of the levelized cost of energy [2]. In this context, condition monitoring based on supervisory control and data acquisition (SCADA) systems has become a cost-effective strategy for preventive maintenance, enabling accurate early-stage fault detection, diagnosis, and warning [3].

Conventional fault diagnosis methods mainly rely on dedicated signal analysis techniques, such as vibration analysis [4,5,6], infrared thermography [7], and acoustic detection [8,9]. Although these techniques are effective for assessing the health of mechanical components, they show clear limitations when applied to faults in the increasingly complex electrical, electronic, and hydraulic systems of modern wind turbines. In addition, methods including spectral analysis and wavelet transform [9] face difficulties in feature extraction when dealing with high-dimensional SCADA data characterized by nonlinear correlations. SCADA systems record key time-series variables related to the overall health of wind turbines, including temperature measurements, environmental conditions, and energy conversion indicators. Consequently, increasing attention has been directed toward statistical modeling and intelligent learning techniques, which are capable of mining fault-related representations from high-dimensional historical SCADA observations.

Early studies have demonstrated the effectiveness of multivariate statistical methods for fault identification [10]. For instance, clustering algorithms such as K-means have been applied to automatically identify abnormal patterns in SCADA data [11]. However, clustering-based methods often require significant prior knowledge for parameter setting and result interpretation. Moreover, they often struggle to capture complex latent nonlinear relationships when dealing with high-dimensional feature combinations.

Intelligent learning methods have substantially reduced the dependence on human expertise. Vidal et al. [12] and Han et al. [13] employed support vector machines (SVMs) to effectively distinguish wind turbine operating states and establish relationships among operational parameters. Zhao et al. [14] compared the performance of artificial neural networks (ANNs), K-nearest neighbors (KNN), and naive Bayes classifiers for generator fault diagnosis, while Tao et al. [15] demonstrated the high prediction accuracy of machine learning algorithms, such as XGBoost, in gearbox fault prediction. Advancements in deep learning have further empowered models like convolutional neural networks (CNNs) [16,17], deep residual networks (ResNets) [18], and long short-term memory networks (LSTMs) [19,20], which have shown strong capabilities in spatial feature extraction and long-term temporal dependency modeling. In addition, transfer learning has been widely adopted to address diagnostic challenges under varying operating conditions [21,22,23,24].

However, these models still face fundamental challenges in practical applications. First, wind turbine faults are often incipient and progressively developing; consequently, supervised learning suffers from severe class imbalance between normal and faulty samples during training. Second, conventional anomaly detection methods, such as unsupervised and semi-supervised techniques, can issue early warnings but struggle to capture high-order nonlinear correlations within high-dimensional data. This renders accurate root cause diagnosis challenging when multiple subsystems suffer concurrent faults [25,26,27]. Furthermore, existing models relying solely on Transformer or CNN architectures tend to prioritize either temporal relations or local features and thus cannot fully reflect the diverse operating states of wind turbines.

To address the above challenges, this study proposes a Multi-Scale Memory Attention Network (M^2^ANet) for 30-min-ahead wind turbine fault prediction using conventional SCADA data. The main contributions of this work are summarized as follows:

(1) A multi-scale progressive dilated convolution module is developed. By employing parallel kernels and progressive dilation, it expands the shallow receptive field to capture both fast- and slow-varying SCADA dynamics, thereby enhancing early-stage fault feature extraction.
(2) A temporal-spectral feature extraction module is designed to model complex temporal evolutions and variable correlations. Specifically, a dual-path memory network captures global degradation trends and local variations; a channel attention mechanism models nonlinear correlations among high-dimensional SCADA variables to locate concurrent subsystem faults; and an auxiliary STFT branch extracts periodic spectral features masked in the raw time-series.
(3) A predictive entropy regularization loss based on the L_1_-distance target constraint is proposed to mitigate model overconfidence caused by class imbalance. By dynamically bounding the predicted probability distribution, this loss regularizes the decision boundary, thereby improving the diagnostic accuracy for minority fault categories.

## 2. SCADA Data Analysis

The experimental data used in this study were collected from the SCADA system of 40 operational 5-MW doubly fed induction generator wind turbines in a wind farm located in Fujian, China. The dataset consists of three independent subsets: operational measurements, status records, and daily fault logs. The data covers an eight-month period from June 2025 to January 2026. Considering the physical relationships among different variables and the reliability of sensor measurements, the model inputs are defined as multidimensional feature vectors, as listed in Table 1.

To capture the temporal evolution of incipient faults and to enlarge the training dataset, an overlapping sliding-window strategy was applied to segment the raw SCADA time series. For each fault event, the 30 min of data immediately preceding the fault occurrence are selected as the analysis interval. This specific duration is determined based on practical wind farm operation and maintenance records, which indicate that the early anomalous precursors for most subsystem faults dominantly manifest and evolve within a window of 20 to 35 min before a protective shutdown. Selecting a 30-min horizon ensures comprehensive coverage of these precursor dynamics while providing sufficient lead time for onsite operators to perform remote diagnostic verification. Within this interval, the sequence was divided using a window length of L = 300 s and a stride of S = 100 s. Each fault event could generate up to 16 subsequences, and every valid subsequence was assigned the same class label as the corresponding fault event.

To prevent data leakage and sample overlap between different sets, we partition the raw continuous time-series data chronologically into training, validation, and testing sets before applying the sliding window. Specifically, we split the original data into three contiguous, non-overlapping periods. The sliding window with a 200 s step overlap is then applied independently within each subset. Consequently, no training samples share overlapping segments with validation or testing samples, eliminating cross-dataset leakage.

Normal-condition samples were extracted only from fault-free wind turbines. In this study, a turbine was considered fault-free if no alarm or fault record of any type was triggered during the complete calendar month of data acquisition. This criterion was adopted to reduce the influence of potential sub-healthy operating states on model discrimination.

**Figure 1:**
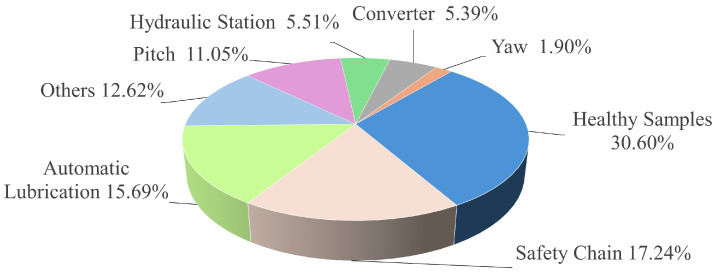
Dataset Composition and Class Distribution.

All samples were processed using a consistent data-cleaning procedure. Any candidate sliding window containing missing values was regarded as invalid and removed from the dataset. In addition, an active under-sampling strategy was adopted to roughly control the ratio of normal samples to fault samples at 0.5:1. The final dataset contains 17,244 valid samples, covering normal operating conditions and seven typical fault categories. As shown in Figure 1, the samples are highly unevenly distributed across different classes. Notably, yaw system faults account for only 1.9% of the total dataset. This severe class imbalance reflects the actual operating conditions of wind farms and poses greater challenges to the generalization ability and robustness of the proposed model.

## 3. Multi-Scale Memory Attention Network

**Figure 2:**
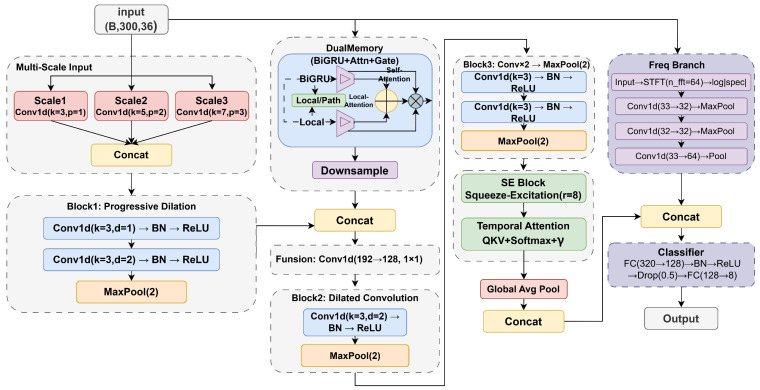
Model structure of the proposed M^2^ANet.

Conventional deep learning models often struggle to effectively capture the evolution patterns of rare faults in wind turbines, owing to the strong noise contamination, multi-scale spatiotemporal correlations, and extreme class imbalance present in monitoring signals. To address these challenges, this study proposes a novel Multi-Scale Memory Attention Network (M^2^ANet). By integrating a dual-memory module with progressive dilated convolution, the proposed model enables efficient extraction of multi-scale temporal features. The overall topology of M^2^ANet is illustrated in Figure 2.

### 3.1. Multi-Scale Progressive Dilated Convolution Module

SCADA data contains both fast-varying signals, such as active power and wind speed, and slow-varying signals, such as ambient temperature and oil temperature. Therefore, a convolution operation with a single kernel size is insufficient to characterize these heterogeneous temporal patterns simultaneously. In addition, the model is required to capture the long-term environmental evolution reflected in eight months of wind turbine operating data. To this end, this branch employs parallel convolutional layers with kernel sizes of k=3, 5, and 7 to model the frequency-response characteristics of different physical variables. Specifically, k=3 is used to capture short-term transient fluctuations in active power, whereas k=7 is more suitable for extracting slowly varying patterns from variables such as oil temperature.

Unlike conventional designs that use a uniform dilation strategy, Block 1 adopts a progressive dilation scheme with d=1→2. This design effectively alleviates the gridding effect commonly introduced by deep dilated convolutions. As a result, the receptive field can be enlarged while preserving the critical high-frequency transient features in SCADA signals, including those caused by fluctuations in sensor sampling behavior. The progressive dilation strategy also avoids the information loss that may occur when large dilation rates are directly applied in shallow layers.

After the initial encoding stage, Block 2 employs consecutive convolutional layers with a fixed dilation rate of d=2. This stage plays a key role in rapidly expanding the receptive field, enabling the model to identify medium- and long-term operating patterns and thus perceive deviations in the system-level performance state of the wind turbine. According to the cumulative receptive field calculation, the receptive field (RF) of this layer reaches 308 time steps, providing global coverage of the complete input sequence of 300 steps.

As the temporal dimension is progressively downsampled through the pooling layers, Block 3 returns to standard convolution with d=1. The purpose of this stage is to aggregate the accumulated temporal representations into a high-dimensional latent space and ultimately extract highly discriminative “fault fingerprints” from the raw time-series signals. It should be noted that an excessively large dilation rate, such as d=4, may make the extracted features overly sparse and consequently degrade model performance. Therefore, using small dilation rates in shallow layers and moderate dilation rates in deeper layers provides a more effective design strategy.

### 3.2. Adaptive Dual-Path Memory Module

The Dual-Path Memory module comprises three components: a global memory path, a local memory path, and a gated fusion mechanism. Its forward propagation process is detailed in Algorithm 1. Specifically, the module consists of two parallel processing branches: 1. Global memory path: This path employs a Bidirectional Gated Recurrent Unit (BiGRU) combined with a self-attention mechanism. The BiGRU captures bidirectional long-range temporal dependencies within the sequence, while the self-attention mechanism overcomes distance limitations to enhance the model’s perception of global contextual information. 2. Local memory path: Discarding recurrent structures, this path directly utilizes a self-attention mechanism to process linearly projected features. It focuses on capturing fine-grained variations and local patterns within short temporal windows. To balance the feature contributions from different temporal scales, a gated fusion mechanism is introduced. This mechanism adaptively generates weight parameters based on the dynamic characteristics of the input data, performing a weighted sum on the global and local features to produce the module’s output.
**Algorithm 1** Forward Propagation of the Dual-Path Memory Module**Input:** Input feature tensor $X\in R^{B\times T\times C_{in}}$, where *B* is the batch size, *T* is the number of time steps, and $C_{in}$ is the number of input channels.**Output:** Fused feature representation $Y\in R^{B\times T\times d_{model}}$, where $d_{model}$ is the hidden dimension of the model.  ▹*—Phase 1: Global Memory PathwayThe triangle symbol in the algorithms is just a visual marker to indicate a stage or section, and it has no special mathematical meaning, so no additional explanation is needed. The removal of italics and the emdash formatting are also acceptable. —*$H_{bi}\leftarrow \mathrm{BiGRU}\left(X\right)$                        ▹ BiGRU extracts long-range temporal dependencies, $H_{bi}\in R^{B\times T\times 2d_{g}}$$H_{norm}\leftarrow \mathrm{LayerNorm}\left(H_{bi}\right)$                                ▹ Layer normalization for training stability$A_{global}\leftarrow \mathrm{MultiHeadSelfAttn}\left(H_{norm},h=4\right)$                          ▹ Multi-head self-attention captures global context$g_{out}\leftarrow H_{norm}+A_{global}$                        ▹ Residual connection to preserve original temporal information                                             ▹*—Phase 2: Local Memory Pathway—*$L_{proj}\leftarrow \mathrm{Linear}\left(X,\mathrm{out}\_\dim=d_{l}\right)$                        ▹ Linear projection to align input to model dimension$L_{norm}\leftarrow \mathrm{LayerNorm}\left(L_{proj}\right)$                                         ▹ Layer normalization$A_{local}\leftarrow \mathrm{MultiHeadSelfAttn}\left(L_{norm},h=4\right)$                           ▹ Focus on local patterns and fine-grained variations$l_{out}\leftarrow L_{norm}+A_{local}$                                              ▹ Residual connection                                              ▹*—Phase 3: Gated Adaptive Fusion—*$Z_{concat}\leftarrow \mathrm{Concat}\left(g_{out},l_{out}\right)$                                    ▹ Concatenate global and local features$\alpha \leftarrow \sigma (\mathrm{Linear}\left(Z_{concat}\right))$                                 ▹ Sigmoid generates gating weight α∈[0,1]$Y\leftarrow \alpha ⊙g_{out}+\left(1-\alpha \right)⊙l_{out}$                        ▹ Weighted fusion: dynamic balance between global and local paths**return** *Y*

To align and facilitate concatenation with the output of Block 1, the output of the Dual Memory module is first downsampled. Subsequently, a 1 × 1 convolutional layer is applied to fuse the concatenated features, compressing the channel dimension from 192 to 128 to eliminate redundant information. The fused features then sequentially pass through a dilated convolution block (Block 2) and a standard convolution block (Block 3) to extract deeper representations. Finally, these features are fed into the Squeeze-and-Excitation (SE) and Temporal Attention module for further refinement.

### 3.3. Squeeze-and-Excitation and Temporal Attention Module

Wind turbine SCADA data contains multiple monitoring channels; however, the contributions of different variables to various fault categories vary significantly. Concurrently, within a single time window, not all temporal segments possess equal discriminative value. To address this, a cascaded module consisting of Squeeze-and-Excitation (SE) [28] and Temporal Self-Attention mechanisms is introduced following deep convolutional aggregation. The forward propagation process is detailed in Algorithm 2. This algorithm primarily comprises two core steps: first, channel weights are calculated via the SE block to perform adaptive channel recalibration on the input features (Lines 6–9); subsequently, the Temporal Self-Attention mechanism is utilized to capture long-range temporal dependencies, yielding the final enhanced features through a residual connection (Lines 10–11). This process effectively enables the model to achieve dual-dimension feature focusing in both the channel and temporal spaces.
**Algorithm 2** Forward Propagation of the SE and Temporal Attention Module  1:**Input:** Input feature tensor $X\in R^{B\times T\times C}$, where *B* is the batch size, *T* is the number of time steps, and *C* is the number of channels; learnable scaling parameter γ∈R (initialized to 0).  2:**Output:** Optimized feature tensor $Y\in R^{B\times T\times C}$.▹*—Phase 1: SE Module (Channel Attention)—*  3:$Z_{global}\leftarrow \mathrm{GlobalAvgPool}\left(X\right)$
▹ Squeeze temporal dimension: $R^{B\times 1\times C}$
  4:$W_{excitation}\leftarrow \sigma \left(W_{2}\left(\delta \left(W_{1}\left(Z_{global}\right)\right)\right)\right)$
▹ Generate channel-wise attention weights  5:${\tilde{X}}_{channel}\leftarrow X⊗W_{excitation}$
▹ Channel feature recalibration▹*—Phase 2: Temporal Attention Mechanism—*  6:$Q,K,V\leftarrow \mathrm{Linear}({\tilde{X}}_{channel})$
▹ Linear projection (equivalent to 1×1 convolution)  7:$A_{scores}\leftarrow \frac{QK^{T}}{\sqrt{d_{k}}}$
▹ Compute scaled dot-product attention scores  8:$A_{weights}\leftarrow \mathrm{Softmax}\left(A_{scores}\right)$
▹ Normalize along temporal dimension  9:$Y_{temporal}\leftarrow A_{weights}V$
▹ Aggregate temporal context information▹*—Phase 3: Progressive Residual Fusion—*10:$Y\leftarrow \gamma \cdot Y_{temporal}+{\tilde{X}}_{channel}$
▹ Progressive residual connection scaled by γ
11:**return** *Y*

### 3.4. Time–Frequency Analysis Module

To supplement the representation capability of the main time-domain network, the model introduces a frequency-domain auxiliary feature extraction module to perform time–frequency transformation and spectral modeling on the input sequence. Considering that the raw input is a multivariate SCADA sequence, we first perform mean aggregation across the feature channels to obtain a one-dimensional time-series representation: $x_{f}\in R^{B\times 300}$. Subsequently, the Short-Time Fourier Transform (STFT) is applied to obtain the time–frequency representation within local time windows. Its definition is formulated as:

(1) $$S\left(t,\omega \right)=\sum_{n=0}^{N-1}x\left(n\right)w\left(n-t\right)e^{-j\omega n}$$

where x(n) denotes the sequence value of a single sample at the *n*-th time step, *n* is the time sampling index, *N* represents the analysis window length, w(·) is the window function (the Hann window is adopted in this study), *t* denotes the temporal position of the short-time window, ω is the angular frequency, and S(t,ω) represents the complex spectral coefficient at the temporal position *t* and frequency ω. To mitigate the dynamic range variance of the spectral magnitude and improve numerical stability, a log-spectrogram representation is further constructed as:

(2) $$S_{log}=\log\left(|S\left(t,\omega \right)|+\epsilon \right)$$

where ϵ is an extremely small constant to prevent taking the logarithm of zero. After STFT processing, the frequency-domain input feature $S_{log}\in R^{B\times 33\times T_{f}}$ is obtained, where $T_{f}$ denotes the number of temporal frames after STFT. On this basis, a one-dimensional convolutional network is employed to extract features from the spectrogram. The frequency-domain convolutional branch progressively uncovers local frequency patterns and cross-frequency band coupling relationships through multiple convolutional and pooling layers, eventually yielding the frequency-domain feature vector via adaptive average pooling:

(3) $$z_{freq}=\mathrm{FreqConv}\left(S_{log}\right)\in R^{B\times 64}$$

This frequency-domain branch is capable of extracting periodic fault features from the time–frequency representation that are challenging to model directly by the time-domain network, forming an effective complement to the backbone time-domain features.

The model extracts time-domain and frequency-domain features from the backbone time-domain branch and the auxiliary frequency-domain branch, respectively. Subsequently, these two types of features are concatenated along the feature dimension to form a joint feature representation, which is then fed into a fully connected classifier to perform fault category prediction. The classifier utilizes a Softmax function at its end to output the predicted probability for each category. During the model training phase, the cross-entropy loss function is employed as the optimization target to update the network parameters by minimizing the prediction errors between the predicted categories and the ground-truth labels.

### 3.5. Loss Function Optimization Strategy Based on Predictive Entropy Regularization

Although the standard Cross-Entropy (CE) loss effectively drives the model to fit the training labels, it often induces over-confident predictions. Mathematically, this phenomenon manifests as the predicted probability distribution converging towards a one-hot distribution, resulting in minimal predictive entropy. In the context of wind turbine data characterized by noise and fluctuating operating conditions, such over-confidence undermines the model’s generalization capability and degrades the calibration of fault predictions. Furthermore, it leads to either overly conservative or excessively aggressive predictions for minority fault classes with limited samples.

To address the challenge of extreme class imbalance in SCADA-based wind turbine fault diagnosis (e.g., yaw faults accounting for merely 1.9%), this paper employs a loss function incorporating predictive entropy regularization. By establishing a target entropy threshold, this function constrains the uncertainty of the prediction distribution within a reasonable and robust range.

Assume that the output probability distribution of the model for the *i*-th sample is $P_{i}=\left[p_{i1},p_{i2},\ldots ,p_{iC}\right]$, where *C* denotes the number of fault categories (C=8, including one normal class and seven fault classes). The Shannon entropy of this distribution is defined as

(4) $$H\left(P_{i}\right)=-\sum_{c=1}^{C}p_{ic}\log\left(p_{ic}\right),$$

where $H\left(P_{i}\right)\in \left[0,\log C\right]$. When $H(P_{i})=0$, the model prediction is completely confident, which may indicate overfitting. In contrast, when $H(P_{i})=\log C$, the model prediction is fully uncertain, suggesting that discriminative features have not been effectively learned. Therefore, a target entropy $H_{\mathrm{target}}$ is defined as a specified proportion of the maximum entropy, representing the desired stable state of the model prediction:

(5) $$H_{\mathrm{target}}=\alpha \cdot \log C,\;\alpha \in \left(0,1\right).$$

The total loss function $L_{\mathrm{total}}$ is formulated as a weighted combination of the standard cross-entropy loss $L_{\mathrm{CE}}$ and an entropy-deviation penalty term:

(6) $$L_{\mathrm{total}}=L_{\mathrm{CE}}+\lambda \cdot R\left(H\left(P_{i}\right),H_{\mathrm{target}}\right).$$

Here, λ is a positive hyperparameter that controls the trade-off between the classification objective and the entropy regularization term. The function R(·) denotes a distance metric that measures the deviation between the prediction entropy $H(P_{i})$ and the target entropy $H_{\mathrm{target}}$.

For the choice of regularization form, a common practice is to use the squared-error formulation based on the $L_{2}$ distance. However, in wind turbine SCADA-based fault detection, the data often contain random disturbances and outliers. Under such conditions, an $L_{2}$ penalty produces gradients that grow linearly with the deviation, which may cause excessive fluctuations during the early stages of training and interfere with optimization of the primary classification objective. For this reason, the penalty term in this study is constructed using the $L_{1}$ distance:

(7) $$R=\frac{1}{N}\sum_{i=1}^{N}\left|H\left(P_{i}\right)-H_{\mathrm{target}}\right|,$$

where *N* is the total number of samples.

Because the derivative of the absolute deviation remains constant at 1 (except at zero), this formulation prevents the regularization strength from becoming excessively large when the deviation increases, thereby avoiding gradient explosion caused by over-penalization. When $H\left(P_{i}\right)<H_{\mathrm{target}}$, the term produces a negative gradient that drives the model to reduce overconfidence and improve generalization. In contrast, when $H\left(P_{i}\right)>H_{\mathrm{target}}$, it yields a positive gradient that encourages the model to enhance the certainty of feature extraction. This bidirectional adjustment mechanism centered on $H_{\mathrm{target}}$ allows the model to converge toward a prediction distribution with improved calibration. Accordingly, the final loss function is written as

(8) $$L_{\mathrm{total}}=-\frac{1}{N}\sum_{i=1}^{N}\sum_{c=1}^{C}y_{ic}\log\left(p_{ic}\right)+\lambda \cdot \frac{1}{N}\sum_{i=1}^{N}\left|H\left(P_{i}\right)-H_{\mathrm{target}}\right|.$$

Here, $y_{ic}$ denotes the ground-truth label of the *i*-th sample for class *c*, typically represented using one-hot encoding, where the true class position is 1 and all others are 0. The term $p_{ic}$ denotes the predicted probability that the *i*-th sample belongs to class *c*.

### 3.6. Performance Evaluation

To comprehensively and objectively evaluate the performance of the proposed M^2^ANet in wind turbine fault prediction, Accuracy, Macro Precision, Macro Recall, and Weighted F1-score are adopted as evaluation metrics.

First, the basic elements of the confusion matrix are defined. For class *i*, $TP_{i}$, $TN_{i}$, $FP_{i}$, and $FN_{i}$ denote the numbers of true positives, true negatives, false positives, and false negatives, respectively. In this study, there are *C* classes in total, where C=8, including one normal class and seven fault classes. For each class *i*, the precision ($P_{i}$), recall ($R_{i}$), and F1-score ($F1_{i}$) are calculated as follows:

(9) $$Precision_{i}=\frac{TP_{i}}{TP_{i}+FP_{i}},$$

(10) $$Recall_{i}=\frac{TP_{i}}{TP_{i}+FN_{i}},$$

(11) $$F1_{i}=\frac{2\times Precision_{i}\times Recall_{i}}{Precision_{i}+Recall_{i}}.$$

Considering the class imbalance in the dataset, the weighted F1-score is calculated as

(12) $$F1=\sum_{i=1}^{C}\left(\frac{N_{i}}{N}\times F1_{i}\right),$$

where *N* denotes the total number of samples, and $N_{i}$ is the number of samples belonging to class *i*.

Accuracy measures the overall proportion of correctly classified samples and is defined as

(13) $$Accuracy=\frac{\sum_{i=1}^{C}TP_{i}}{N}.$$

This metric provides an intuitive indication of the overall classification performance of the model. However, in class-imbalanced scenarios, it can be dominated by majority classes and is therefore used only as a supplementary reference metric in this study.

## 4. Results and Discussion

### 4.1. Model Comparison Experiments

To validate the effectiveness of the proposed $M^{2}$ANet model, we compared it with ten baseline models spanning four categories: (1) CNN-based models including BasicCNN, DeepCNN [29], ResNet1D [30], and DenseNet1D [31]; (2) RNN-based models including BiLSTM [32] and Attention-LSTM [33]; (3) hybrid models including CNN-LSTM [34] and Transformer [35]; and (4) the temporal convolution model WaveNet [36]. We also include a traditional machine learning baseline, SVM [37].The detailed hyperparameter settings of all baseline models are presented in Table 2.

To ensure the reliability of the results, all metrics reported in the figures and tables are averaged over 10 independent runs. The main performance metrics are summarized in Table 3. As shown in Table 3, $M^{2}$ANet achieves a macro recall of 94.04%, outperforming the second-best model, DeepCNN (90.06%), by 3.98 percentage points. This indicates that the proposed model has the lowest missed detection rate across different fault categories, making it particularly suitable for early warning requirements in faults such as yaw system failures. By contrast, BiLSTM, SVM, and AttentionLSTM show relatively poor performance, suggesting that conventional recurrent neural networks alone are insufficient to capture deep temporal dependencies in SCADA data. In comparison, $M^{2}$ANet effectively extracts more discriminative features through progressive multi-scale dilated convolution.

**Figure 3:**
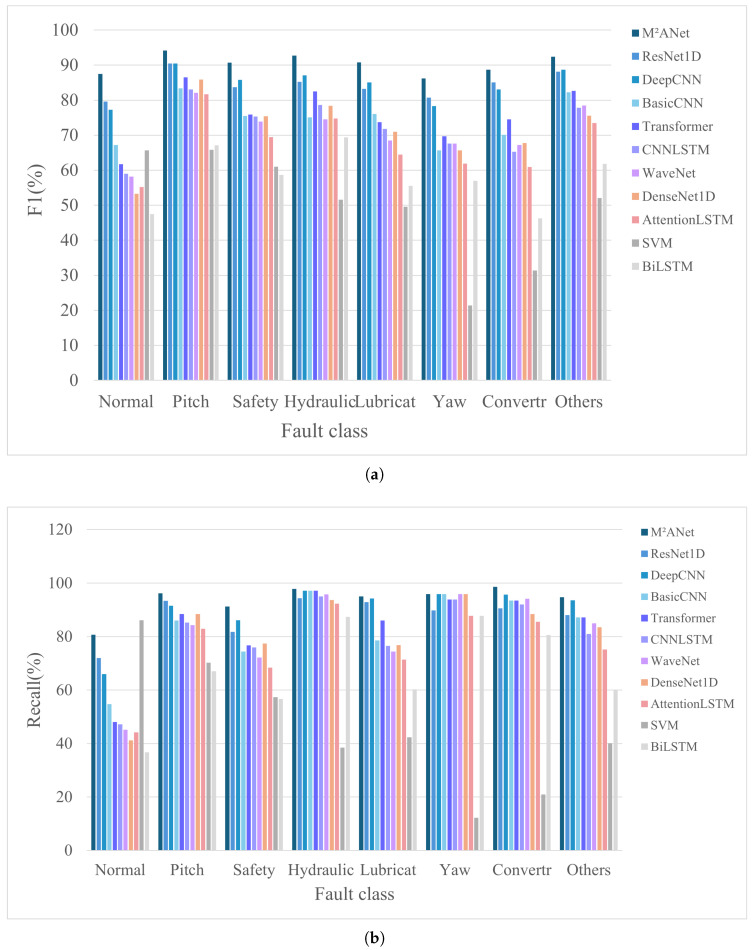
Diagnostic performance comparison of different models across eight fault classes: (**a**) F1-score, and (**b**) Recall.

To further investigate the performance of the proposed method across different fault categories, Figure 3 compares the prediction results of different models for each fault type. As shown in Figure 3, the proposed $M^{2}$ANet consistently outperforms the ten baseline models in terms of both F1-score and recall across almost all fault categories. In particular, Figure 3a shows that $M^{2}$ANet achieves F1-scores above 90% for most critical faults, including pitch, safety, and hydraulic system faults, with the highest F1-score of 94.2% obtained for pitch faults. These results indicate that the proposed model achieves a strong balance between precision and recall, effectively reducing both false alarms and missed detections.

For highly noisy and low-sample fault categories, such as yaw faults and converter faults, conventional baselines such as SVM and BiLSTM show limited effectiveness. For example, the F1-score of SVM on yaw fault classification is only 21.4%. In contrast, $M^{2}$ANet maintains strong predictive performance, achieving an F1-score of 86.2% for yaw faults and a recall of 98.5% for converter faults. The F1-scores of ResNet1D and DeepCNN remain stable in the range of 75–85%, indicating that one-dimensional convolutional architectures are well suited to capturing local spatiotemporal patterns in wind turbine monitoring time-series data. In contrast, CNNLSTM and BiLSTM show relatively weak performance. This is mainly because conventional recurrent gated structures are more susceptible to interference from high noise levels and long-period temporal variations, which can weaken their ability to model long-term dependencies.

As shown in Figure 3b, the recall of $M^{2}$ANet for the normal class is noticeably lower than that for the fault categories, and is even slightly lower than that of SVM. This is mainly because the model is highly sensitive in boundary decision-making. To preserve strong capture capability for incipient faults, the decision boundary is intentionally set to be relatively conservative. As a result, a small proportion of normal samples in transitional operating conditions or with moderate fluctuations are classified as slight anomalies, leading to a modest increase in the false alarm rate for the normal class and a slight reduction in normal-class recall.

**Figure 4:**
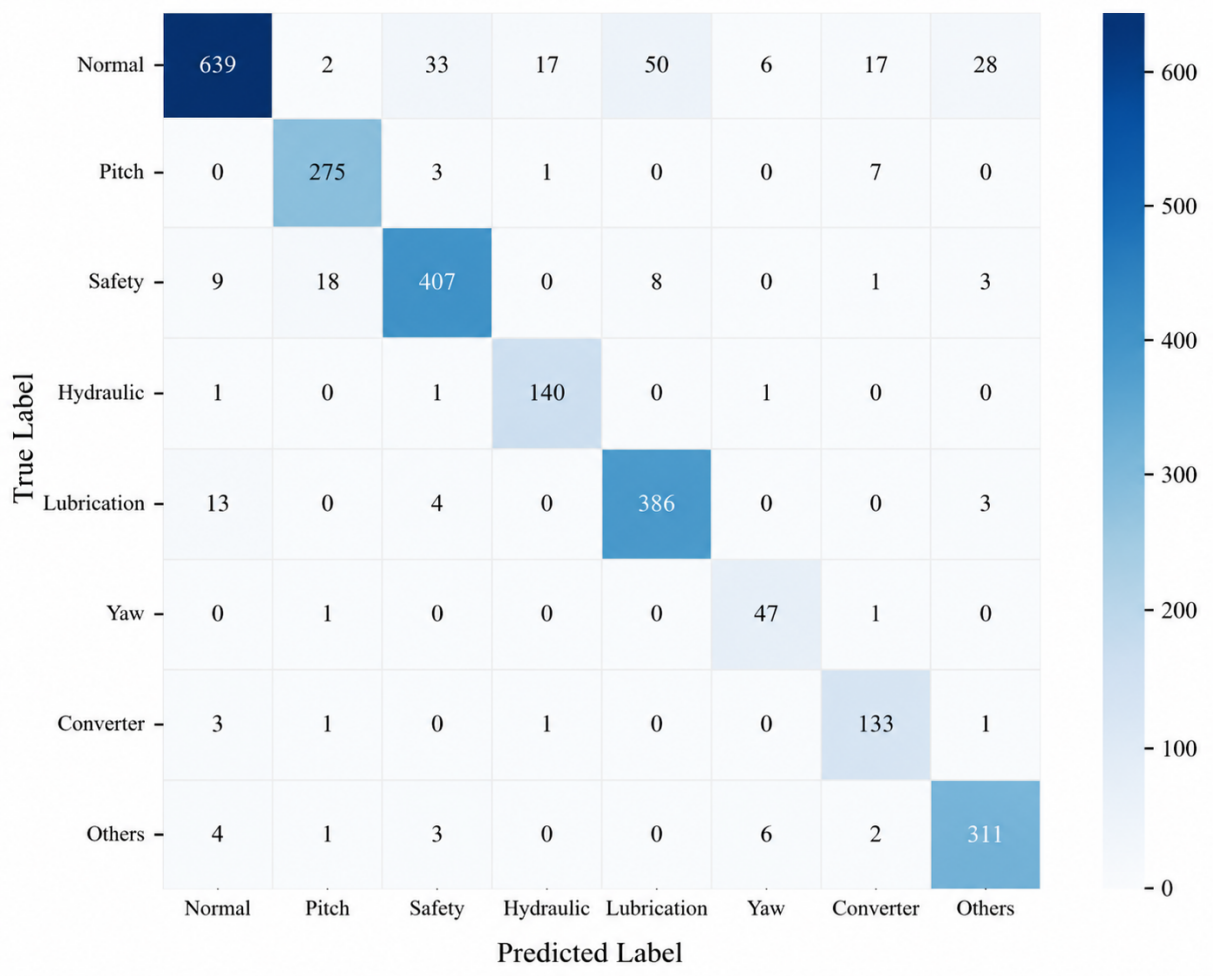
Confusion matrix on the test set of M^2^ANet.

The misclassification behavior of $M^{2}$ANet was further analyzed using the confusion matrix, as shown in Figure 4 For pitch, hydraulic, yaw, and converter faults, the diagonal entries account for a large proportion of the total samples, while the off-diagonal entries remain very small, mostly 0 or 1. This indicates that the model can effectively identify the characteristics of small-sample fault categories, demonstrating high reliability in the recognition of critical turbine faults.

In contrast, normal operation, safety chain faults, lubrication system faults, and other faults exhibit a certain degree of misclassification. The most prominent error occurs when normal samples are classified as lubrication system faults, suggesting that the feature boundary between normal operation and early-stage lubrication faults is relatively ambiguous. It is also worth noting that 2% of safety chain faults and 3.2% of lubrication system faults are misclassified as normal operation. This further indicates that lubrication system degradation is typically a progressive process, where early-stage fault signatures are weak and can be easily absorbed by the model into normal background noise.

### 4.2. Extended Performance Analysis

#### 4.2.1. Sensitivity Analysis of λ

**Figure 5:**
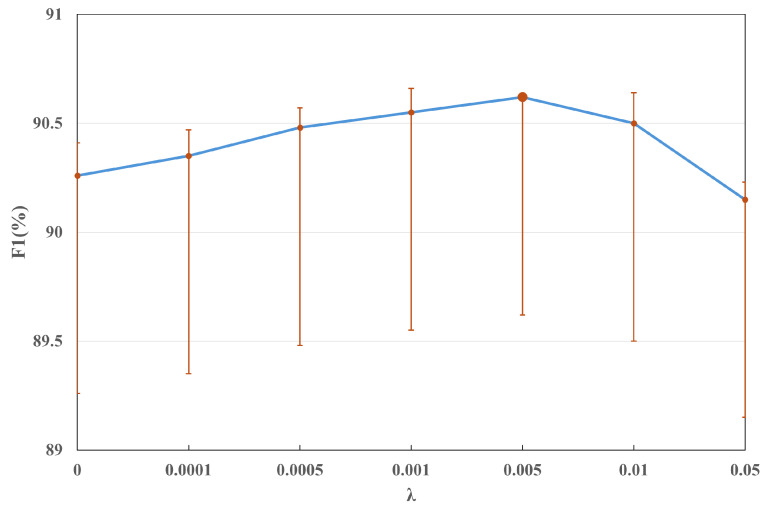
Effect of λ on F1 Score.

To further investigate the influence of the hyperparameter λ on the diagnostic performance of the proposed model, a sensitivity analysis was conducted by varying λ within a predefined range. Figure 5 illustrates the variation in F1-score under different values of λ. As shown in Figure 5, the proposed M^2^ANet achieves the best performance when λ=0.005, yielding an F1-score of 90.62%. When λ is increased to 0.05, the performance slightly declines. This phenomenon suggests that an excessively large λ may cause the entropy regularization term to dominate the optimization process, thereby weakening the contribution of the primary diagnostic objective.

In addition, when λ=0, without entropy regularization, M^2^ANet achieves an F1-score of 90.26%. In comparison, introducing entropy regularization with λ=0.005 improves the F1-score to 90.62%, indicating that the entropy regularization term contributes to a further improvement in diagnostic performance.

#### 4.2.2. Expected Calibration Error Analysis

To further evaluate the reliability of the model’s confidence estimation, the Expected Calibration Error (ECE) is employed to measure the consistency between predicted confidence and actual correctness. A lower ECE indicates better calibration performance. The model trained with the standard cross-entropy loss yields an ECE of 0.0351, whereas the introduction of entropy regularization reduces the ECE to 0.0209, corresponding to a relative decrease of 40.5%. This result indicates that the probability calibration quality of the model is significantly enhanced.

This improvement can be mainly attributed to the entropy penalty term in the loss function, which imposes a constraint on the predicted probability distribution and thus alleviates the overconfidence problem to a certain extent. Consequently, the confidence scores generated by the model are more consistent with the actual diagnostic correctness. In practical fault prediction tasks, reliable confidence estimation is crucial for risk-aware decision-making and early warning.

#### 4.2.3. Performance Analysis Across Different Prediction Horizons

**Figure 6:**
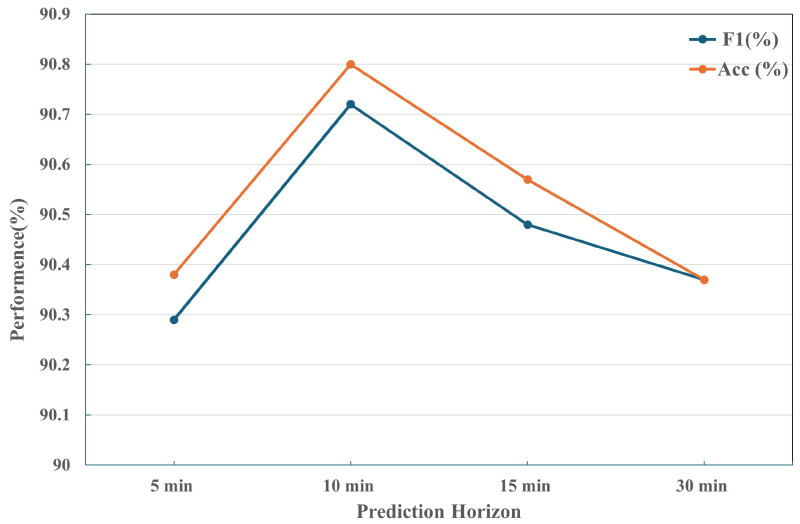
Performance Comparison of F1 Score and Accuracy Across Different Prediction Horizons.

To evaluate the model’s early warning capability across different time horizons prior to fault occurrence, we conducted a comprehensive assessment of its classification performance using a test set generated via an overlapping sliding window approach. The analysis covers prediction lead times ranging from 5 to 30 min. As illustrated in Figure 6, the M^2^ANet model demonstrates consistent stability and accuracy across all prediction horizons, maintaining an F1 score consistently above 90%.

Notably, the model achieves its peak performance at the 10-min horizon with an F1-score of 90.72%, reflecting the turbine’s physical degradation process. Within the 30-min pre-fault window, the turbine transitions through three successive stages: latent degradation from 30 to 20 min, a critical state from 20 to 10 min, and a pre-failure state in the final 10 to 5 min. At the 10-min mark, which corresponds to the critical state, physical anomalies like thermal accumulation and structural vibrations become prominent, providing the most distinct SCADA fault signatures. At the 30-min horizon, although early precursors are subtle and obscured by normal operational variations, the model still sustains a robust F1-score of 90.3%. The ability of $M^{2}$ANet to maintain a stable F1-score above 90% across this entire period demonstrates its capacity to capture precursor information from the onset of degradation, meeting industrial warning requirements.

#### 4.2.4. Evaluation of Computational Efficiency

In terms of parameter efficiency, as shown in Table 4, $M^{2}$ANet contains 0.923 million trainable parameters, which is at a moderate scale compared with the baseline models. Although its parameter size is larger than that of the lightweight WaveNet, the substantial improvement in classification accuracy justifies the additional computational cost. During practical inference, the average processing time on the test set containing 2588 samples is approximately 0.12 s per sample, which fully satisfies the requirements of real-time online monitoring in wind farms.

### 4.3. Ablation Study

To evaluate the contribution of each module in M^2^ANet, we conducted ablation experiments by removing one module at a time while keeping all other settings unchanged. Each variant was evaluated using three random seeds, and the reported weighted F1 scores are presented as mean ± standard deviation. Table 5 lists the weighted F1 scores, relative F1 changes compared to the full model, and the number of trainable parameters. After removing the frequency-domain branch across all modules, the weighted F1 score dropped to 88.99%, a decrease of 1.23 percentage points. This result highlights the importance of frequency-domain information in SCADA-based fault prediction. Removing the temporal attention module and the SE module individually led to decreases of 0.54 and 0.49 percentage points in the F1 score, respectively. These results indicate that both temporal feature selection and channel-wise recalibration contribute to extracting fault-discriminative representations from multivariate SCADA signals. The removal of the progressive dilated convolution module resulted in a 0.10 percentage point drop in F1 score, demonstrating its role in modeling long-range temporal dependencies.

The Dual Memory module is primarily responsible for memorizing temporal fault features and retaining historical anomaly information. Removing this module alone leads to a significant decrease of 1.68 percentage points in the F1-score, demonstrating its crucial role in enhancing the model’s detection accuracy. In contrast, while the Multi-Scale Convolution module yields only a minor performance degradation when removed individually, it exhibits a significant complementary effect with the Dual Memory module. Specifically, when both modules are removed simultaneously, the overall performance drops by 2.2 percentage points, which is notably larger than the degradation caused by removing the Dual Memory module alone. This indicates that the Multi-Scale Convolution module provides an additional synergistic contribution of approximately 0.52% in mining multi-scale local fault features, effectively supplementing local detailed information when temporal memory features are absent.

## 5. Conclusions

To address the high complexity of wind turbine SCADA data, the difficulty of extracting discriminative fault features, the scarcity and imbalance of fault samples, and the tendency of deep learning models to become overconfident in classification, this paper proposes a novel fault prediction model, $M^{2}$ANet, with a prediction entropy regularization term incorporated into the loss function. Comparative experiments were conducted against ten mainstream machine learning and deep learning models.

The experimental results show that the proposed $M^{2}$ANet achieves superior performance across all evaluation metrics. Specifically, the fault prediction accuracy reaches 90.73%, and the weighted F1-score reaches 90.62%. Compared with the classical ResNet1D and Transformer models, the F1-score is improved by approximately 6.84 and 17.22 percentage points, respectively, confirming the advantage of the proposed model in capturing temporal patterns from wind turbine SCADA signals. In addition, by integrating prediction entropy regularization into the loss function, the model effectively alleviates the overconfidence problem induced by standard cross-entropy loss. This conservative training strategy not only improves the overall classification accuracy, but also enhances the robustness of the model when dealing with fault classes that have ambiguous decision boundaries.

The proposed model also demonstrates clear advantages in handling class-imbalanced fault data. For yaw system faults, which account for only 1.9% of the total samples, $M^{2}$ANet achieves a recall of 95.92%. This property is of practical importance for wind farm operation and maintenance, as it can substantially reduce the risk of missed detections for severe faults in key turbine components.

While maintaining high diagnostic accuracy, $M^{2}$ANet maintains a reasonable model size, with approximately 0.923 million trainable parameters. Its efficient inference speed, approximately 0.12 s per sample, satisfies the requirements of real-time fault diagnosis in remote monitoring centers of wind farms, demonstrating strong potential for practical engineering deployment.

Despite its high prediction accuracy and computational efficiency, a key limitation of this study is that the operational data are obtained from a single wind farm with uniform turbine models. Consequently, the performance of the model under different geographical terrains, varying wind regimes, or different turbine designs warrants further validation. To address this limitation, future research will extend the applicability of the proposed model to cross-wind-farm, cross-region, and cross-turbine scenarios.

## Figures and Tables

**Table 1:** Descriptions of SCADA parameters.

Subsystem	Parameters	Unit
Pitch System	Blade pitch angle setting (1/2/3)	°
	Blade pitch angle actual (1/2/3)	°
Yaw & Anemometry	Wind speed	m/s
	Yaw rate	°/s
	Yaw error	°
Drive Train & nacelle	Rotor speed 1	r/min
	Rotor speed 2	r/min
	Nacelle fore-aft vibration (X)	-
	Nacelle left-right vibration (Y)	-
Hydraulics & Braking	High-speed shaft brake pressure	bar
	Hydraulic station system pressure	bar
	Yaw system hydraulic pressure	bar
Converter System	Converter measured generator torque	N·m
	Converter measured generator speed	r/min
	Converter torque reference	N·m
	Converter module temperature	°C
	Active power	kW
Power Quality	Phase voltage L1N/L2N/L3N	V
	Phase current 1/2/3	A

**Table 2:** Hyperparameter settings of all baseline models.

Model	Key Hyperparameters	Dropout
BasicCNN	Conv layers = 3, channels = [64, 128, 256], kernel = 3, MaxPool(2)	–
DeepCNN	VGG blocks = 3, conv/block = 2, channels = 64 → 128 → 256, kernel = 3, MaxPool(2)	0.5 (Fully Connected Layer)
BiLSTM	layers = 2, hidden = 128, bidirectional	0.3
CNN-LSTM	CNN: channels = 64, kernel = 3, 2 × MaxPool(2); LSTM: layers = 2, hidden = 128, bidirectional	0.3
Attention-LSTM	LSTM: layers = 2, hidden = 128, bidirectional; Attention: Linear(256 → 128→ 1) + tanh	0.3
Transformer	$d_{\mathrm{model}}=128$, heads = 8, layers = 2, FFN dim = 512	0.1
ResNet1D	stages = [64, 128, 256], blocks/stage = 2, initial kernel = 7	0.1
WaveNet	channels = 64, kernel = 2, dilations = [1, 2, 4, 8, 16, 32, 64]	0.5 (Fully Connected Layer)
DenseNet1D	growth rate = 16, dense blocks = 3, layers/block = 3, initial channels = 32	0.5 (Fully Connected Layer)
SVM	kernel = RBF, C=10, γ = scale, probability = True	–

**Table 3:** Overall performance comparison of different models.

Model	Accuracy	Weighted F1	Precision	Recall
M^2^ANet	90.73%	90.62%	89.25%	94.04%
ResNet1D	83.93%	83.78%	82.15%	87.87%
DeepCNN	84.12%	83.77%	81.08%	90.06%
BasicCNN	74.65%	74.29%	70.42%	83.45%
Transformer	74.38%	73.40%	72.23%	83.87%
CNNLSTM	71.21%	70.46%	68.40%	80.91%
WaveNet	70.17%	69.28%	67.39%	80.88%
DenseNet1D	70.05%	68.71%	67.50%	80.68%
AttentionLSTM	66.54%	65.90%	64.19%	75.97%
SVM	59.20%	57.20%	67.71%	45.97%
BiLSTM	56.34%	55.97%	55.24%	67.12%

**Table 4:** Parameter count and training time comparison of different models.

Model	Type	Parameters	Time
M^2^ANet	Deep Learning	923,817	324 s
ResNet1D	Deep Learning	1,098,056	72 s
DeepCNN	Deep Learning	424,456	57 s
BasicCNN	Deep Learning	133,192	44 s
Transformer	Deep Learning	418,824	296 s
CNNLSTM	Deep Learning	615,560	64 s
WaveNet	Deep Learning	70,408	73 s
DenseNet1D	Deep Learning	51,906	67 s
AttentionLSTM	Deep Learning	600,329	145 s
SVM	Machine Learning	N/A	67 s
BiLSTM	Deep Learning	599,176	133 s

Note: “N/A” indicates that the parameter count is not applicable for the SVM model, as it does not have trainable parameters in the same sense as deep learning models.

**Table 5:** Ablation study and contribution ranking of different modules.

Removed Module	Weighted F1 (%)	Drop (%)	Parameters
Full M^2^ANet	90.22 ± 0.29	–	923,817
Time–Frequency Module	88.99 ± 0.13	+1.23	911,049
Temporal Attention	89.68 ± 0.20	+0.54	922,481
SE	89.73 ± 0.16	+0.49	907,145
Progressive Dilated Convolution	90.13 ± 0.30	+0.10	923,817
DualMemory	88.54 ± 0.36	+1.68	639,657
Multi-Scale+DualMemory	88.02 ± 0.07	+2.20	632,745

## Data Availability

The data that support the findings of this study are available from the corresponding author upon reasonable request.
